# Supplementary material for: Assessment of variability in the plasma 7k SomaScan proteomics assay
Source: Sci Rep. 2022 Oct 13;12:17147. doi: 10.1038/s41598-022-22116-0 (PMC9561184; doi:10.1038/s41598-022-22116-0)
Supplement: Supplementary file 6 — Supplementary Information 6. [file 41598_2022_22116_MOESM6_ESM.pdf]

Supplementary Information

Assessment of Variability in the Plasma 7k SomaScan Proteomics Assay

Julián Candia[1,*], Gulzar N. Daya[2], Toshiko Tanaka[1], Luigi Ferrucci[1], and Keenan A. Walker[2]

[1]Translational Gerontology Branch, National Institute on Aging, National Institutes of Health, Baltimore, MD 21224, USA
[2]Laboratory of Behavioral Neuroscience, National Institute on Aging, National Institutes of Health, Baltimore, MD 21224, USA
*julian.candia@nih.gov

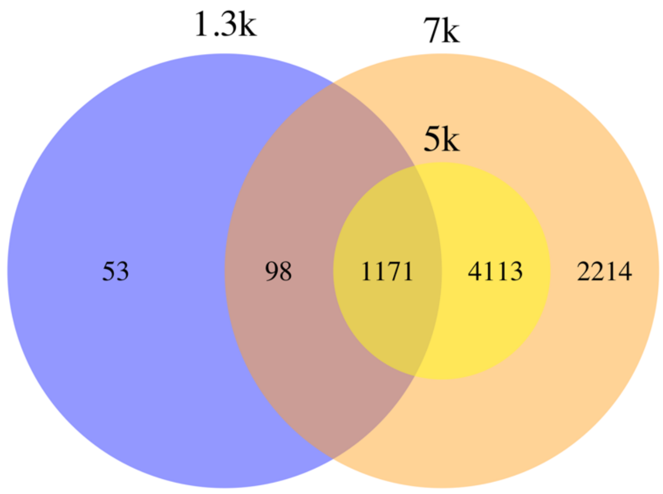

Supplementary Figure 1. Venn diagram showing the SOMAmer overlap (based on the unique "SeqId" identifiers) between the 1.3k (v3), 5k (v4), and 7k (v4.1) SomaScan assays. For more details, see **Supplementary Data 1**.

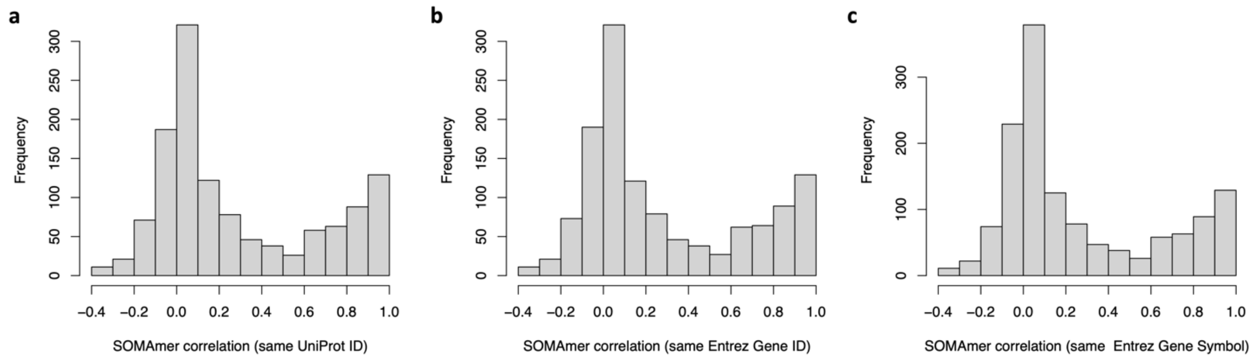

Supplementary Figure 2. Frequency distributions of pairwise Pearson's correlation of log_{10}(RFU) across 1,799 experimental samples between SOMAmers that share the same annotated target proteins according to: **(a)** UniProt ID, **(b)** Entrez Gene ID, and **(c)** Entrez Gene Symbol. For more details, see **Supplementary Data 5**.

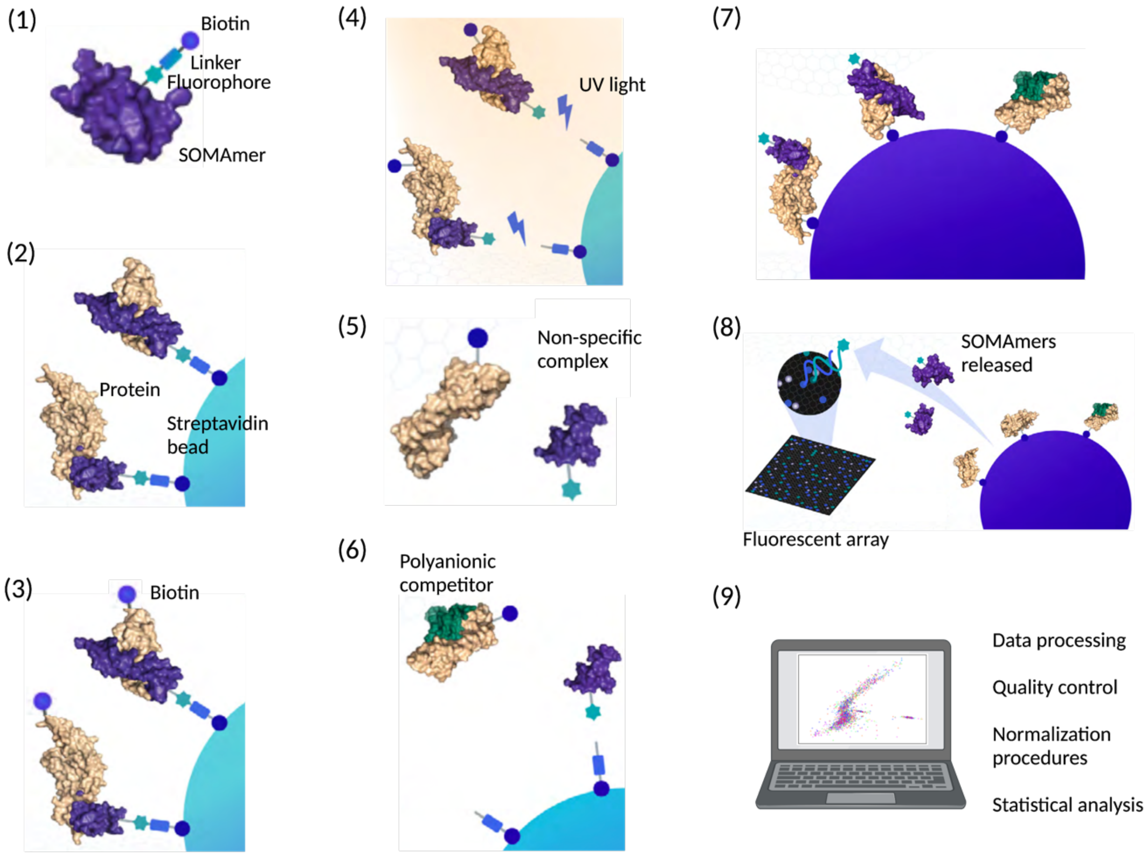

Supplementary Figure 3. Workflow of the assay. (1) SOMAmers are synthesized with a fluorophore, photocleavable linker, and biotin; (2) diluted samples are incubated with dilution-specific SOMAmers bound to streptavidin beads; (3) unbound proteins are washed away, and bound proteins are tagged with biotin; (4) UV light breaks the photocleavable linker, releasing complexes back into solution; (5) non-specific complexes dissociate while specific complexes remain bound; (6) a polyanionic competitor is added to prevent rebinding of non-specific complexes; (7) biotinylated proteins (and bound SOMAmers) are captured on new streptavidin beads; and (8) after SOMAmers are released from the complexes by denaturing the proteins, fluorophores are measured following hybridization to complementary sequences on a microarray chip. Upon completion of all experimental steps, (9) the bioinformatic analysis proceeds. Adapted from SomaLogic's Technical Note SL00000572 and created with BioRender.
